# Lymphangioma Formation Following Hyaluronic Acid Injection for Lip Augmentation

**DOI:** 10.7759/cureus.12929

**Published:** 2021-01-27

**Authors:** James Wege, Mohammed Anabtawi, Mike A Blackwell, Alan Patterson

**Affiliations:** 1 Oral and Maxillofacial Surgery, Castle Hill Hospital, Hull, GBR; 2 Oral and Maxillofacial Surgery, Chesterfield Royal Hospital, Chesterfield, GBR; 3 Oral and Maxillofacial Surgery, Leeds General Infirmary, Leeds, GBR; 4 Oral and Maxillofacial Surgery, Rotherham National Health Service Foundation Trust, Rotherham, GBR

**Keywords:** lip augmentation, lymphangioma, filler, cosmetic, complication, hyaluronic acid, aesthetics, adverse reaction, facial swelling

## Abstract

Administration of hyaluronic acid (HA) filler for aesthetic lip augmentation is a routine and common procedure with a low rate of adverse reactions. This case report documents an extremely rare complication of lip augmentation with HA leading to the development of lymphangiomas.

Lymphangiomas are uncommon hamartomas of the lymphatic system. Although usually congenital, they can be acquired due to trauma, inflammation, or lymphatic blockage. They may be in the deep or superficial tissues, with superficial forms being either lymphangioma circumscriptum or acquired lymphangioma, also referred to as lymphangiectasia. Acquired lymphangiomas are typically formed by blockage of lymphatic drainage leading to dilation of the lymphatic channels. The diagnosis in our case report is acquired lymphangioma.

A 27-year-old female presented with a two-year history of linear swellings in her upper lip. These lumps followed the line where HA filler had been injected four years earlier. Hyaluronidase had previously been used unsuccessfully to remove these lumps. The patient was treated with surgery to excise the lesions. Five masses were excised, and histopathological analysis displayed the presence of variably ectatic lacunae, lined by cells with CD34 expression, a lymph-vascular-endothelial marker. There were also scattered macrophages with CD68 expression in the interstices. These are typical features of a lymphangioma. The patient was satisfied with the excellent aesthetic and functional outcome.

To our knowledge this is the first case of a lymphangioma following HA lip augmentation. Although rare, this complication can have aesthetic implications for the patient which may require further treatment or surgery to correct.

## Introduction

Hyaluronic acid (HA) is a naturally occurring mucopolysaccharide with a high affinity for water [1]. In humans, it is most apparent in the skin [2], and can account for over half of the total body HA [3]. Most HA is produced endogenously by fibroblasts, among other cell types, and forms part of the extra-cellular matrix where it provides hydration [4]. The use of HA as a cosmetic filler has increased in recent years, especially in the orofacial region, and is generally seen as safe with few complications. We present the first case of its kind of lymphangioma formation following the use of HA for lip augmentation.

## Case presentation

A 27-year-old female was referred to the local Oral and Maxillofacial Surgery (OMFS) department with lumps in her upper lip. Her medical and social history were unremarkable, and there was no history of trauma to the facial region. She gave a four-year history of continuous dryness and tingling in her upper lip, starting shortly after undergoing lip augmentation with HA in both her upper and lower lips. Approximately a year after the injection of the filler she noticed a swelling in her upper lip, which she thought had gradually increased in size since administration. It was unclear whether the swelling was becoming more prominent, or just more obvious due to degradation of the surrounding HA. Two years prior to referral to the OMFS, the patient had hyaluronidase injections to the upper lip, with no noted improvement.

**Figure 1:**
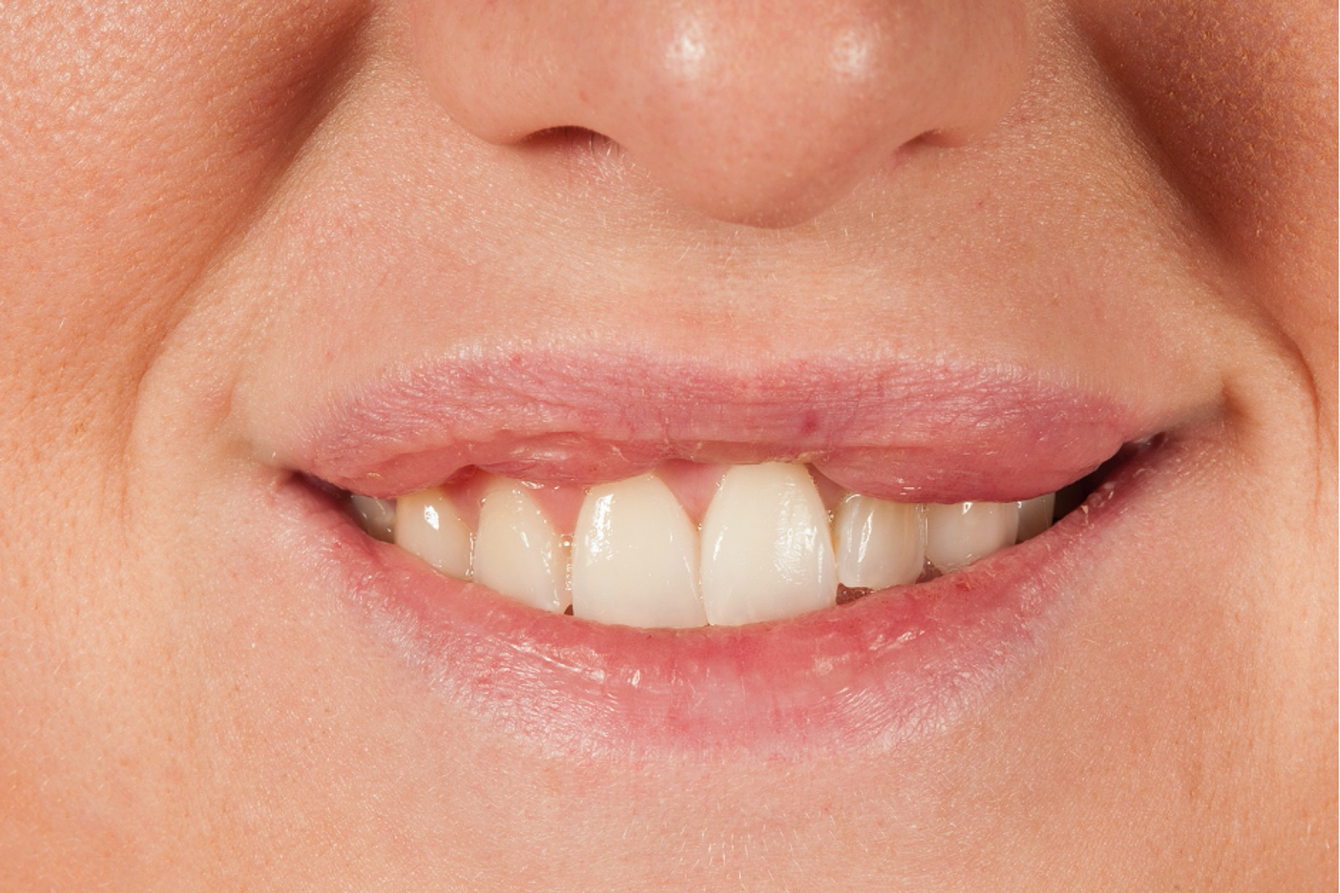
Extra-oral photograph of the patient’s smile. Three separate lumps can be seen on the border of the upper lip, disrupting the smile line.

**Figure 2:**
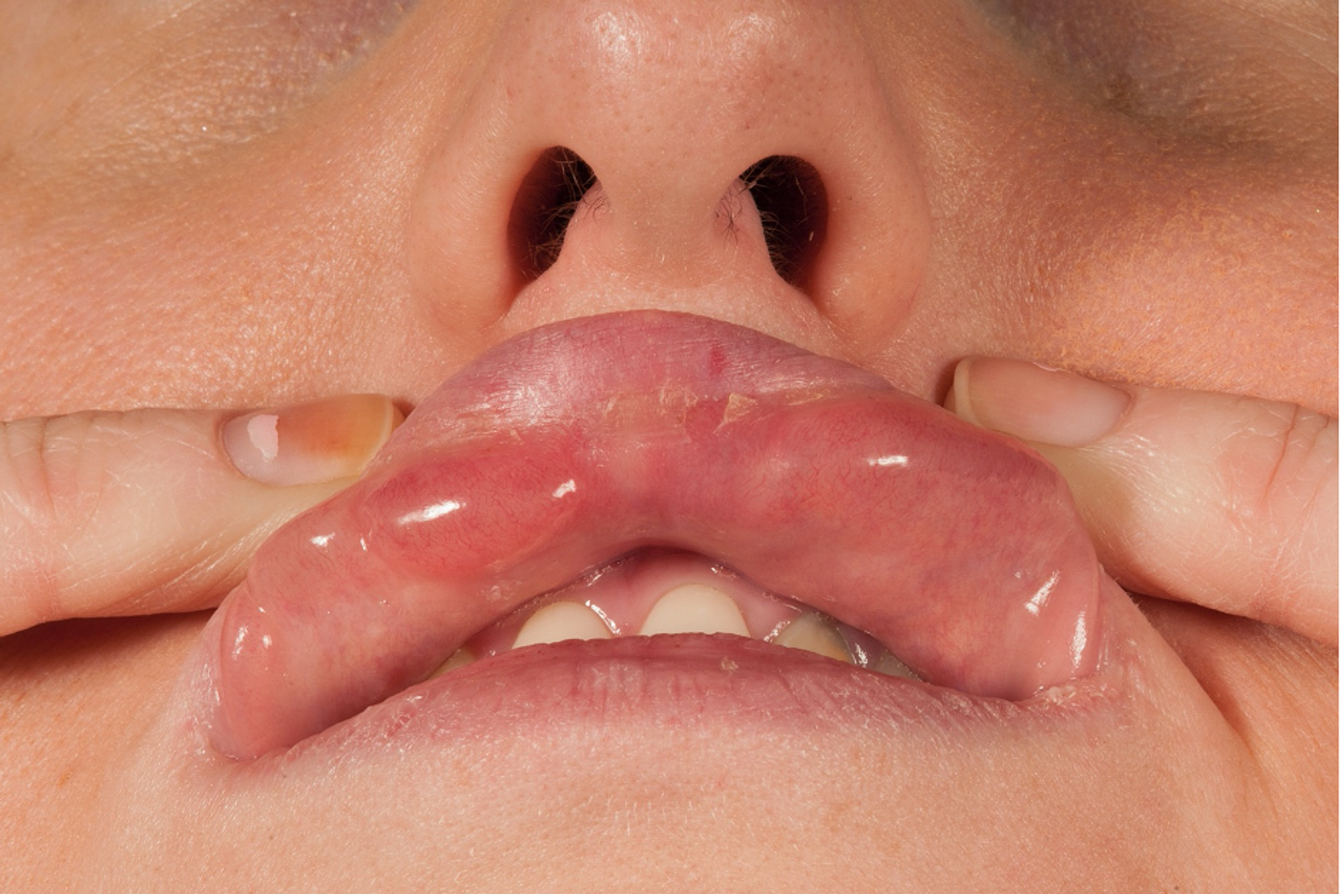
Extra-oral photograph showing multiple well-defined homogenous mucosal colored lumps on the upper lip.

On examination, she had multiple, bilateral, linear, non-tender, and fluctuant swellings in the upper lip. They resembled mucoceles in appearance, although the location of the swellings on the lip did not support this diagnosis. The position of the swellings coincided with the lines of injection of the HA filler. Clinical photographs were taken (Figures 1, 2), and arrangements were made for excision of the swellings.

**Figure 3:**
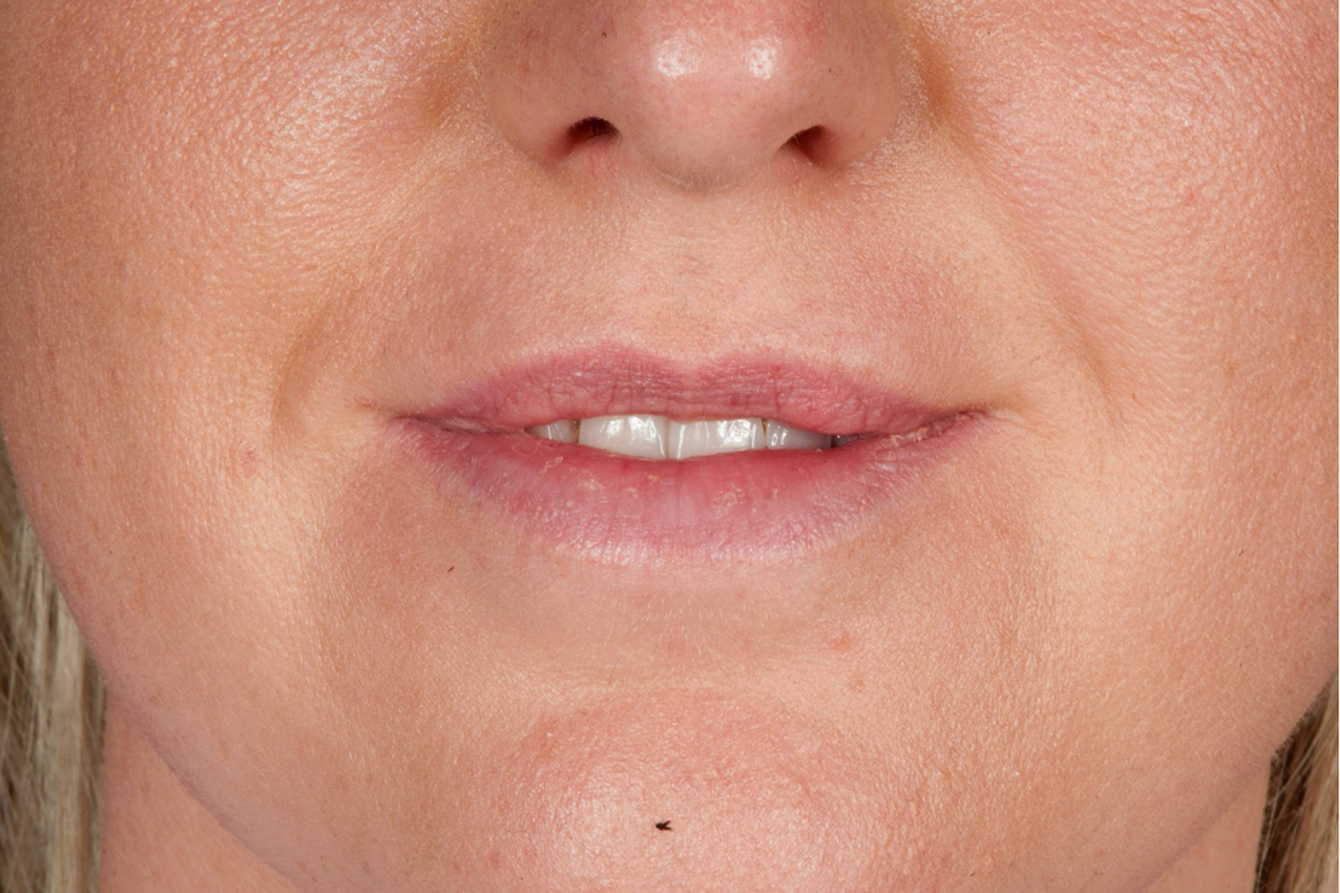
Extra-oral photograph showing the patient’s smile 14 months post-operatively. No residual lumps or scars can be seen.

**Figure 4:**
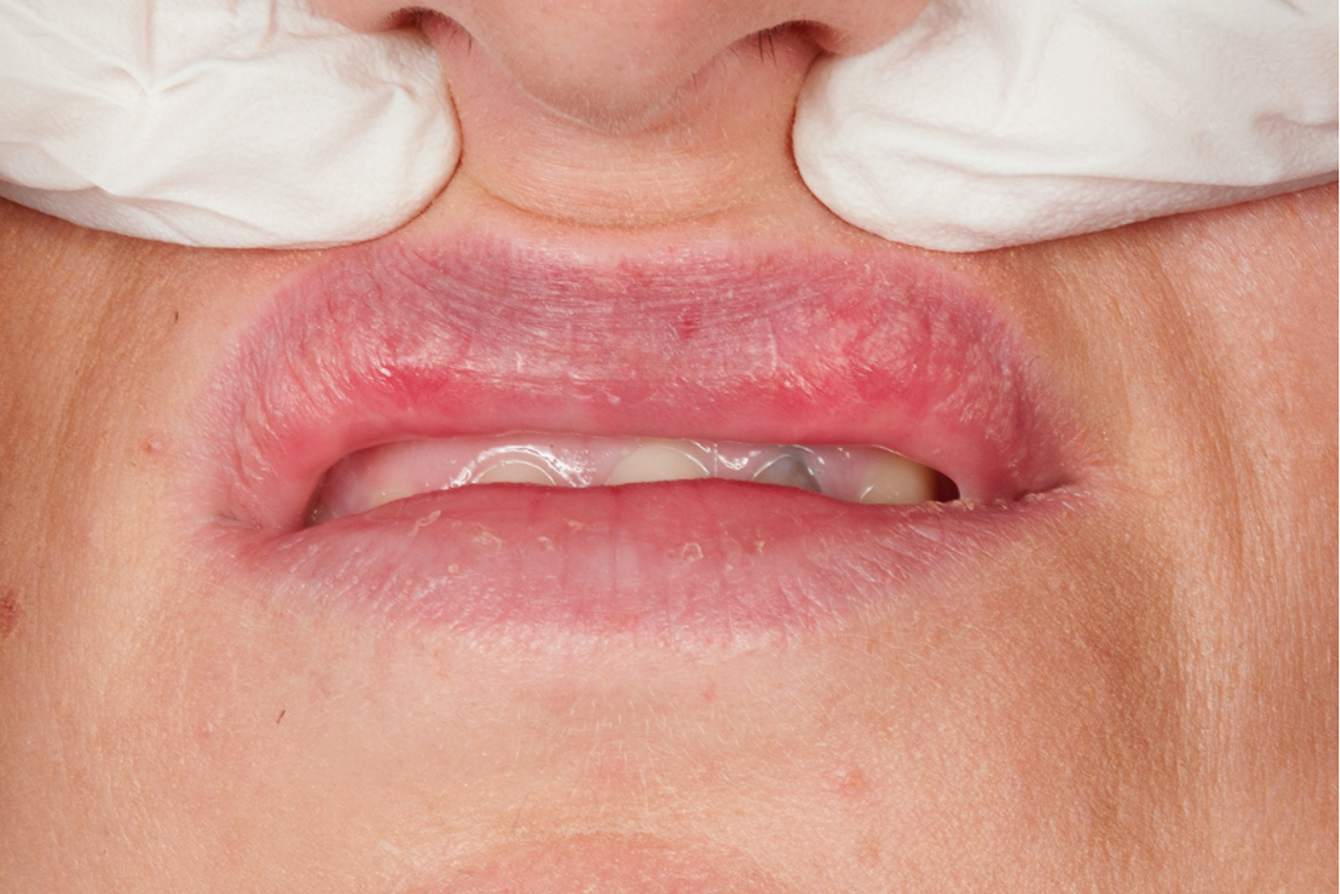
Extra-oral photograph showing the surgical site 14 months post-operatively. There is very slight scarring along the wet and dry junction of the upper lip, with no residual lumps visible.

Excision of the lesions were performed without complication, involving longitudinal incisions on the labial aspect of the upper lip. Five submucosal swellings were identified, dissected, and removed. The deep aspect of the swellings were found to diffusely infiltrate into the underlying orbicularis oris muscle, with no clear plane of excision. The excised tissue was then sent for histopathological analysis. On follow-up at 14 months, the surgical site had healed well with an excellent cosmetic result (Figures 3, 4), and there were no signs of further swellings. The pre-operative dryness and tingling had fully improved after surgery.

**Figure 5:**
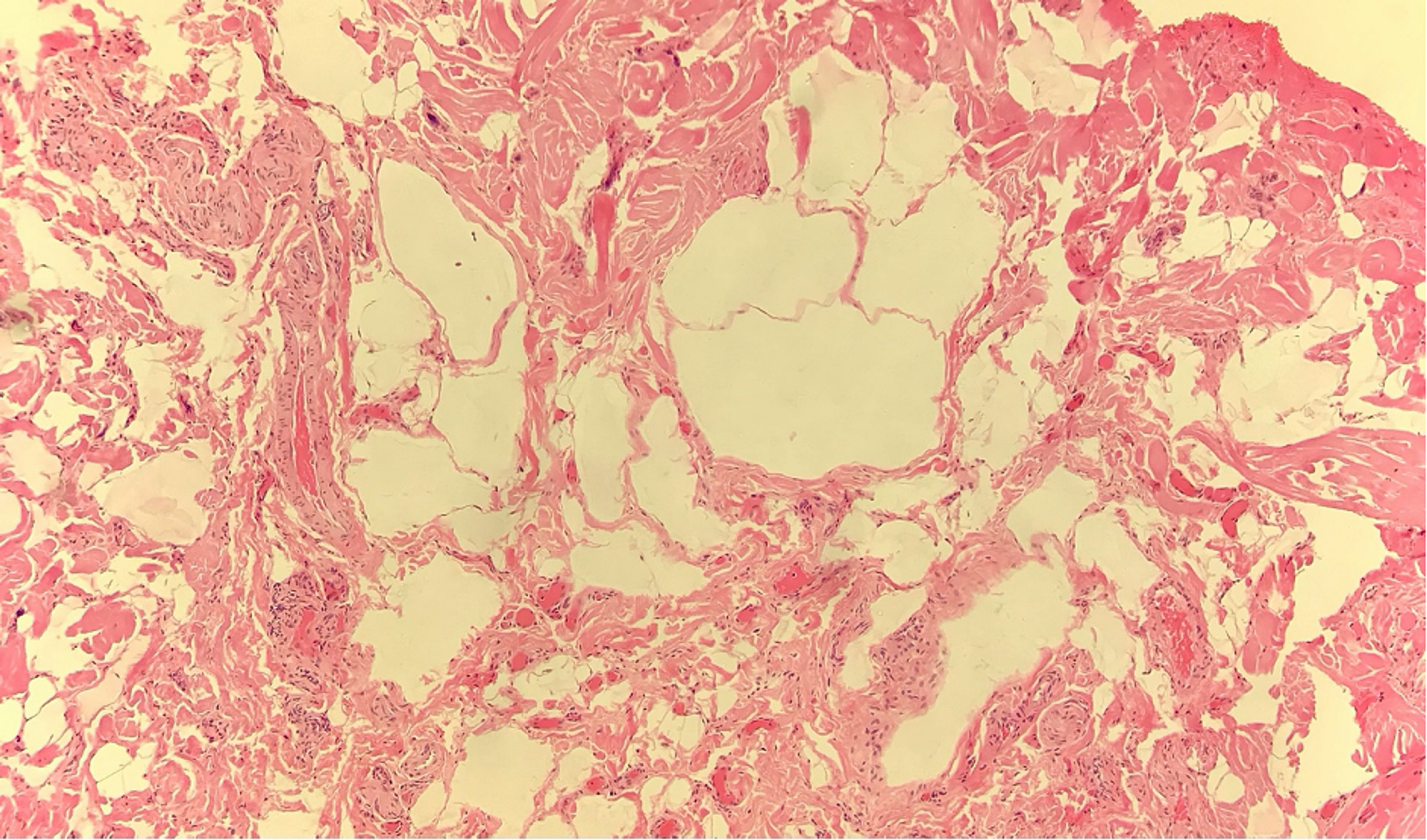
Histology slide with hematoxylin and eosin staining on low magnification showing multiple, variable-sized, vacant-appearing lymphatic lacunae present in the connective tissue.

**Figure 6:**
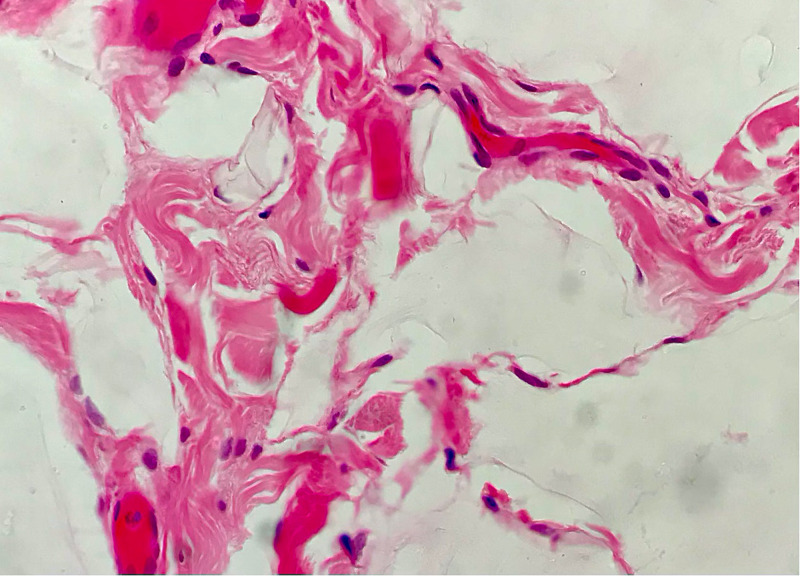
Histology slide with hematoxylin and eosin staining on high magnification showing the endothelial lining with one lacuna apparent.

**Figure 7:**
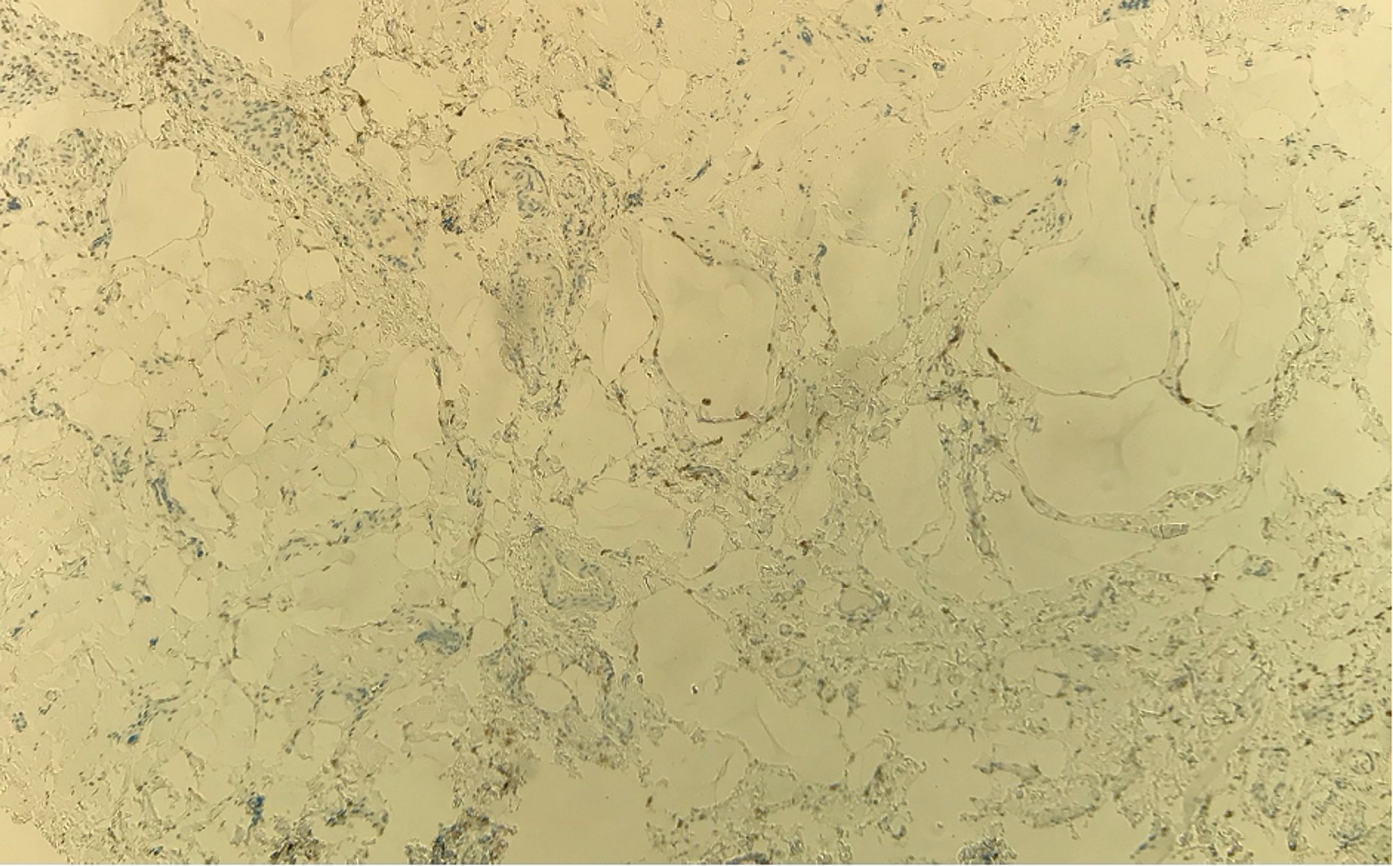
Immunohistochemistry slide on low magnification stained for CD68 (macrophage marker), showing only stray macrophages.

The histopathology report documented five pieces of tissue, the largest measuring 11 mm in diameter. The main pathologic feature was variably ectatic lacunae within the connective tissue and between muscle fascicles (Figures 5, 6). With immunohistochemistry staining, the lacuna lining cells were marked for CD34, with scattered macrophages and CD68 expression in the interstices (Figure 7). The presence of CD34, which is a lymph-vascular-endothelial marker, led to the diagnosis of lymphangioma.

## Discussion

Adverse reactions to HA fillers are rare with a variably reported incidence of 0.5-8% [5-7]. However, nodules caused by HA filler are not rare. They are typically transient and caused by neocollagenesis, leading to the formation of “micronodules” [8]. Other swellings caused by the filler include foreign-body granulomas or sterile abscesses [9]. These swellings typically present soon after the injections, not a year later as in this case. This prolonged time frame led to the consideration of other diagnoses not caused by the filler injection, with mucoceles and minor salivary gland tumors being some of the most common differential diagnoses of labial swellings. Despite nodule formation not being uncommon, lymphangiomas are extremely rare.

Congenital lymphangiomas are uncommon benign hamartomatous lesions found in the lymphatic system. They are typically painless and slow growing, and are a developmental anomaly, usually presenting before the age of five. They are usually found in the head and neck [10]. Superficial lymphangiomas may be either lymphangioma circumscriptum, which is a congenital malformation, or an acquired lymphangioma. Acquired lymphangiomas, also referred to as lymphangiectasia, develop as a result of an abnormality to previously normal lymphatic drainage due to surgery, trauma, malignancy, or radiotherapy [11]. To our knowledge, there are no reported cases of acquired lymphangiomas developing following HA filler injections; however, we suggest that the HA injections interfered with the normal lymphatic drainage mechanism within the upper lip and caused a blockage or reduction in lymphatic drainage leading to the development of the ecstatic lymphatic lacunae.

Lymphangioma after lip filler injection is a diagnosis of exclusion as lumps in the lips are likely to be of other causes and can be self-limiting, so other treatments are usually tried first without success. A typical treatment regime for lumps on the lip after filler injection would be to massage the tissues firmly for several days. If the problem does not resolve, nodules can be pricked with a needle and drained or injected with hyaluronidase if caused by the HA filler injection. Following this, occasionally a trial of intralesional steroids is commenced. In our case, hyaluronidase was attempted before referral. Another peculiarity in this case that led to the consideration of other diagnoses was the considerable length of time that the lesions had remained present without resolution.

Where possible, the definitive treatment of choice for lymphangiomas is surgical excision. Unfortunately, lymphangiomas can be difficult to treat and recurrence rates can be high, with a lower rate of recurrence for superficial lesions [11]. Alternative treatments with sclerotherapy, CO_2_ or Nd-YAG laser, and cryotherapy can also be used, but these provide symptomatic relief rather than definitive options [11].

Although the lymphangiomatous lesions described in the report were harmless, the appearance caused by lumps such as these can cause significant psychological distress, and in this case led to a change in the patient’s behavior and a reduction in social activity. It is reasonable to consider that the aesthetically conscious cohort of patients who seek lip fillers may be particularly distressed by complications such as this. Furthermore, given a common differential diagnosis of labial swellings are minor salivary tumors, excision for histological examination would have been advised to rule out neoplastic disease.

## Conclusions

In summary, lymphangioma is a very rare but possible complication of HA lip filler injection. To our knowledge, this is the first reported case in the literature. For this patient, surgical intervention was required to resolve the complication and rule out other pathology. Surgery resulted in an excellent aesthetic and functional outcome with full resolution of the swelling, dryness, and tingling she initially complained of. Despite the complication that we have described, lip fillers remain a usually safe treatment with limited adverse reactions.
